# Comparison of Tuberculosis Exposure Frequency and Latent Tuberculosis Infection Rates Among Healthcare Workers by Occupational Classification

**DOI:** 10.3390/jcm15031259

**Published:** 2026-02-05

**Authors:** Seran Cheon, Si-Ho Kim, Ji Hong Park, Yu Jin Lee, Jeong Min Shin, Jung Seon Ryu, Nam Sun Hong, Junghwan Cho, Cheon-Hoo Jun, Yu Mi Wi

**Affiliations:** 1 Center for Infection Prevention and Control, Samsung Changwon Hospital, Changwon 51353, Republic of Korea; 2 Division of Infectious Diseases, Department of Internal Medicine, Samsung Changwon Hospital, Sungkyunkwan University School of Medicine, Changwon 51353, Republic of Korea; 3 Division of Endocrinology and Metabolism, Department of Internal Medicine, Samsung Changwon Hospital, Sungkyunkwan University School of Medicine, Changwon 51353, Republic of Korea

**Keywords:** latent tuberculosis, health personnel, disease transmission, infectious, occupational exposure, infection control

## Abstract

**Background:** The Korea Disease Control and Prevention Agency classifies healthcare workers (HCWs) into five groups based on *Mycobacterium tuberculosis* exposure risk and potential transmission impact to guide TB screening strategies. However, data on actual exposure frequency and latent tuberculosis infection (LTBI) incidence across these classifications remain limited. **Methods:** We conducted a retrospective cohort study of HCWs at a tertiary hospital between 2023 and 2024. Baseline LTBI screening was performed for all staff, with annual follow-up testing for Groups 1–4 according to national guidelines. TB exposure history and frequency were investigated and documented by the infection prevention department. LTBI incidence was assessed among HCWs with a negative test in 2023 who underwent repeat testing in 2024. **Results:** Among 2116 HCWs (27.7% male; mean age, 33 years), TB exposure rates increased with higher risk classification (Group 1: 46.9%, Group 2: 31.3%, Group 3: 18.5%, Group 4: 1.2%, Group 5: 0.6%; *p* < 0.001), as did the mean number of exposure events per person (1.39, 0.74, 0.31, 0.01, and 0.01, respectively; *p* < 0.001). The incidence of LTBI was 13/1323 (1.0%). After adjustment, LTBI incidence was associated with ≥2 TB exposure events (adjusted OR, 7.03; 95% CI, 1.87–26.36; *p* = 0.005), but did not differ significantly by group classification. **Conclusions:** This study suggests that occupational classification effectively predicts the frequency of TB exposure among healthcare workers. However, LTBI incidence is more closely associated with multiple TB exposure events rather than occupational classification.

## 1. Introduction

Latent tuberculosis infection (LTBI) refers to a state of persistent immune response to *Mycobacterium tuberculosis* antigens, in which individuals are infected but remain asymptomatic and show no clinical evidence of active tuberculosis and are not infectious [1,2,3]. In low tuberculosis (TB) incidence countries, the World Health Organization recommends LTBI screening for high-risk populations and healthcare workers, who are at increased risk of LTBI and who may pose a risk of TB transmission to vulnerable patients if progression to active TB occurs [2,4,5].

Current guidelines recommend LTBI screening in high-risk populations, including healthcare workers, using either the tuberculin skin test (TST) or interferon-γ release assays (IGRAs) [2,6,7]. The TST is based on a delayed-type hypersensitivity (type IV) immune reaction. Intradermal injection of purified protein derivative, which contains a mixture of *Mycobacterium tuberculosis* antigens, elicits a local T-cell-mediated immune response in individuals who have been previously sensitized through infection and bacille Calmette–Guérin (BCG) vaccination [8]. Although TST has been widely used because of its simplicity and low cost, its specificity is limited in BCG-vaccinated populations because of false positivity [6,8]. In contrast, IGRAs—such as T-SPOT.TB and QuantiFERON assays—measure immune responses to *Mycobacterium tuberculosis*-specific antigens (ESAT-6 and CFP-10) that are absent from BCG strains, resulting in higher specificity [9].

The Republic of Korea had a high incidence of tuberculosis between 2000 and 2010, at approximately 60–80 cases per 100,000 population; however, this incidence has decreased over time to 30.6 cases per 100,000 population in 2023 [10]. In response to this epidemiological burden, and to prevent nosocomial transmission, LTBI testing for healthcare workers has been mandated since August 2017 [11]. Subsequently, the scope and timing of annual LTBI screening were formally defined in a national notification issued in December 2019 under the Tuberculosis Prevention Act. Under these recommendations, healthcare workers were stratified into five groups according to tuberculosis exposure risk, with baseline LTBI screening for all and annual follow-up testing for Groups 1–4 [12]. However, the extent to which this occupational risk group-based screening framework accurately reflects actual TB exposure and LTBI risk among healthcare workers remains insufficiently evaluated. In addition, several factors—such as older age, occupation, and longer duration of employment—have been reported to be associated with LTBI among healthcare workers; however, these associations have largely reflected cumulative exposure and LTBI prevalence rather than the risk of newly acquired infection [13,14,15].

In this context, we aimed to evaluate the association between the five exposure-risk groups and tuberculosis exposure, to determine whether this classification reflects actual LTBI acquisition, and to identify additional factors associated with incident LTBI among healthcare workers.

## 2. Materials and Methods

### 2.1. Hospital Setting and LTBI Screening Protocol

This single-center, retrospective observational study was conducted at Samsung Changwon Hospital, a 750-bed tertiary care hospital. In accordance with the Tuberculosis Prevention Act, healthcare employees were stratified into five exposure-risk groups using an institution-specific classification system adapted from national recommendations (Table 1) [12]. All employees underwent LTBI screening prior to hire. Those classified in Groups 1–4 underwent routine annual tuberculosis screening, which included chest radiography and the T-SPOT.TB assay (Oxford Immunotec Ltd., Abingdon, UK). Given that the BCG vaccination rate in Korea exceeds 97%, the T-SPOT.TB assay was used for LTBI screening [9,16]. T-SPOT.TB assay results were interpreted according to the manufacturer-defined criteria. The result was considered positive if either antigen panel (Panel A or Panel B) showed ≥8 spot-forming cells (SFCs) after subtraction of the Nil control, and negative if both antigen panels showed ≤4 SFCs. Results with 5–7 SFCs in the highest responding antigen well were classified as borderline (equivocal). A test was considered indeterminate if the Nil control spot count exceeded 10 SFCs, or if the positive control spot count was <20 SFCs [17]. All employees diagnosed with LTBI were mandatorily referred to infectious disease physicians and received LTBI treatment; those with contraindications to anti-tuberculosis therapy were closely monitored for tuberculosis occurrence for up to two years. Employees with previously diagnosed LTBI were subsequently followed with chest radiography alone.

At our institution, all TB exposure events were identified through contact investigations. These investigations were conducted by the infection prevention department when a patient with active tuberculosis was admitted without appropriate airborne precautions. A TB contact was defined as an individual who had direct interaction with an index patient while sharing the same enclosed indoor space. Close contact was defined as exposure without appropriate personal protective equipment (PPE) for ≥8 consecutive hours in a single day or for a cumulative duration of ≥40 h. Contacts with exposure durations shorter than these thresholds were classified as casual contacts. The estimated infectious period (risk period for potential transmission) of the index patient was defined as the 3 months preceding the earliest of the following events: onset of TB-related symptoms, sputum acid-fast bacilli smear positivity, or the detection of lung cavitation on chest radiography, in accordance with national guidelines [6,18]. All close contacts underwent baseline chest radiography and a T-SPOT.TB assay, followed by repeat chest radiography and a T-SPOT.TB assay 8–10 weeks after exposure. Casual contacts received education regarding the symptoms of tuberculosis, were monitored for related symptoms, and were instructed to report any TB-related symptoms to their department chief, who would then notify the infection prevention department.

During the study period, owing to the COVID-19 pandemic, the use of respiratory protection equivalent to N95 respirators was mandatory for all healthcare personnel at our institution. However, given the possibility that continuous and appropriate use of respiratory protection could not be fully ensured in real-world clinical settings, all documented tuberculosis exposures during the study period were conservatively classified as casual contacts.

### 2.2. Study Design and Outcomes

We retrospectively reviewed tuberculosis exposure histories documented by the infection prevention department and occupational health records, as well as T-SPOT.TB assay results, among healthcare employees between 2023 and 2024.

The primary outcome was the frequency of documented tuberculosis exposure. The observation period spanned a 2-year period from 2023 to 2024. Exposure frequency was calculated according to occupational tuberculosis risk groups. This outcome was assessed among all healthcare workers (Exposure Cohort).

The secondary outcome was T-SPOT.TB seroconversion. For this analysis, we included only employees who (1) had a negative baseline T-SPOT.TB result and (2) underwent serial T-SPOT.TB screening between 2023 and 2024. For the secondary outcome, the tuberculosis exposure observation period was defined as the interval between consecutive T-SPOT.TB assays. Employees with borderline or indeterminate T-SPOT.TB results were excluded to minimize potential misclassification related to assay variability (Seroconversion Cohort). Factors associated with T-SPOT.TB seroconversion, including occupational TB risk group and exposure frequency, were analyzed.

### 2.3. Statistical Analysis

Descriptive statistics were used to summarize baseline characteristics of the study population. Categorical variables were summarized as frequencies and percentages and compared using Fisher’s exact test when appropriate. Continuous variables were summarized as medians with interquartile ranges (Q1–Q3) and compared across groups using the Kruskal–Wallis test. Occupational TB risk groups were treated as ordered categories. Trends in the proportion of TB exposure across increasing occupational TB risk groups were evaluated using the Cochran–Armitage trend test.

T-SPOT.TB seroconversion was defined as a change from a negative baseline result to a positive follow-up result. Because of the low number of seroconversion events, Firth’s penalized logistic regression was applied to evaluate factors associated with T-SPOT.TB seroconversion, including occupational TB risk group and exposure frequency.

All statistical analyses were performed using R software (version 4.5.0; R Foundation for Statistical Computing, Vienna, Austria). A two-sided *p* value < 0.05 was considered statistically significant.

### 2.4. AI-Assisted Writing and Language Editing

During the preparation of this manuscript, the authors used ChatGPT (OpenAI, GPT-5.2) solely for language polishing and stylistic refinement. All scientific content, interpretations, and conclusions were determined by the authors.

## 3. Results

### 3.1. TB Exposure Across Occupational Risk Groups in Healthcare Workers

**Figure 1:**
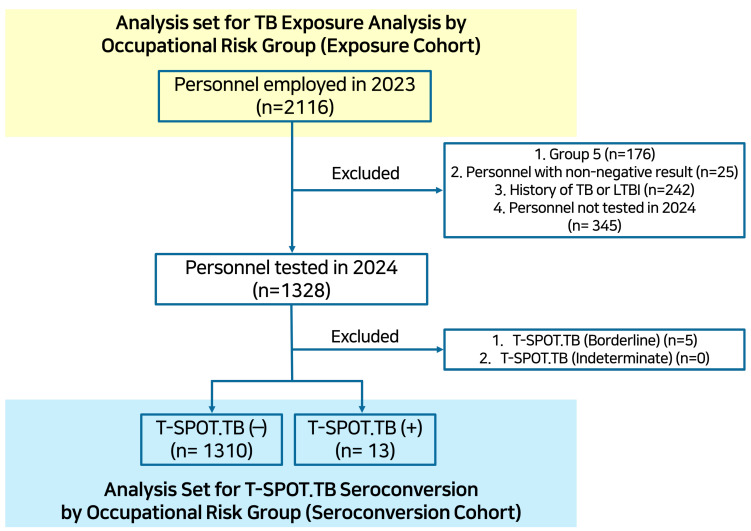
Study Population and Analysis Flow for TB Exposure and Seroconversion. TB, tuberculosis; LTBI, latent tuberculosis infection.

During the study period, a total of 2116 HCWs were included in the analysis (Figure 1, Exposure Cohort). The median age of the study population was 33 years, and 72.3% were female. Overall, 25.3% of HCWs experienced at least one documented TB exposure, and the mean number of TB exposures per individual was 0.62.

Differences in sex and age distributions were observed across occupational risk groups. The proportion of HCWs with TB exposure showed a statistically significant decreasing trend across groups (Group 1, 46.9%; Group 2, 31.3%; Group 3, 18.5%; Group 4, 1.2%; Group 5, 0.6%; *p* < 0.001). A similar decreasing trend was also observed in the mean number of TB exposures per individual (Table 2).

### 3.2. T-SPOT.TB Seroconversion by Occupational TB Risk Group in Healthcare Workers

A total of 1323 HCWs were eligible for the seroconversion analysis (Figure 1, Seroconversion Cohort), among whom the T-SPOT.TB seroconversion rate was 1.0% (13/1323). In univariable analyses, only a TB exposure frequency of ≥2 was associated with a higher incidence of LTBI (0 exposures: 0.7%; 1 exposure: 0.6%; ≥2 exposures: 2.9%; *p* = 0.018). Given the low number of seroconversion events, multivariable analyses were performed using Firth’s penalized logistic regression. In the multivariable analysis, an exposure frequency of ≥2 was independently associated with T-SPOT.TB seroconversion (adjusted odds ratio [aOR], 7.03; 95% confidence interval [CI], 1.87–26.36; *p* = 0.005), along with increasing age (aOR, 1.09 per 1-year increase; 95% CI, 1.03–1.15; *p* = 0.001) (Table 3).

## 4. Discussion

Our study found that occupational TB exposure risk groups among healthcare workers were closely associated with the frequency of documented TB exposure in a tertiary-care hospital setting. In addition, the annual T-SPOT.TB seroconversion rate was approximately 1%. However, occupational risk group classification was not independently associated with T-SPOT.TB seroconversion. Instead, LTBI acquisition was associated with repeated TB exposure at the individual level. These findings suggest that while occupational risk stratification effectively reflects TB exposure risk, the cumulative burden of exposure may be more relevant to LTBI acquisition than occupational category alone.

Several countries implement TB risk stratification for healthcare personnel using occupational health risk assessments, often based on work setting or job-related exposure risk, to guide screening and follow-up strategies. In the United States, baseline TB screening with an individual risk assessment is recommended for all healthcare workers, while routine serial TB testing is not advised in the absence of known exposure or ongoing transmission, except for selected occupational groups at higher risk (e.g., pulmonologists or respiratory therapists) [7]. Italian national guidelines define high occupational TB risk groups based on work setting and job-related exposure, including healthcare workers involved in the care of TB patients, aerosol-generating procedures, and laboratory handling of TB specimens [19]. Therefore, baseline LTBI testing for general HCWs and periodic follow-up testing for specific groups of HCWs have been relatively common strategies; however, the extent and indications for periodic LTBI screening vary according to geographic epidemiology. The results of our hospital suggest that a five-risk group system based on a national guideline is associated with a graded difference in TB exposure frequency, ranging from 0.6% in Group 5 to 46.9% in Group 1.

However, this risk stratification was not directly associated with T-SPOT.TB seroconversion in our study. In systematic reviews of LTBI among healthcare workers, several factors, including both occupational factors (such as previous TB exposure, work location, and engagement in caring for TB patients) and non-occupational factors (such as age), have been suggested to be associated with TST or IGRA conversion [20]. In a study from Thailand, HCWs working in inpatient and outpatient units had a 2.26-fold and 2.10-fold higher risk, respectively, of TST conversion compared with HCWs working in office settings. In addition, a history of TB exposure in the previous year was associated with a 2.46-fold increase in risk [21]. However, another study from Rwanda showed that HCWs were at greater risk of LTBI compared with a control group (school workers), regardless of facility type, work setting, or occupation [22]. It is hard to fully describe the observed discordant results across studies; however, these differences might reflect variations in geographical epidemiology, center-specific characteristics, and evolving infection control strategies guided by national or institutional guidelines [20,23]. In our study, although the frequency of casual exposure varied according to occupational risk group classification, T-SPOT.TB seroconversion was not associated with risk group. Instead, seroconversion was associated with multiple TB exposure events and increasing age in the multivariable model. This apparent discrepancy may be explained by the fact that occupational risk group represents a structural proxy for exposure opportunity rather than a direct measure of individual cumulative exposure. While risk group classification was closely associated with the frequency of TB exposure, LTBI acquisition appears to depend more on repeated or cumulative exposure at the individual level, which is more directly captured by exposure frequency than by occupational category alone. This interpretation aligns with current CDC recommendations, which emphasize individual risk assessment and exposure-based follow-up rather than routine serial testing based solely on occupational category [7]. In our study, increasing age was associated with T-SPOT.TB seroconversion, along with multiple TB exposures. Age is one of the most important factors associated with both LTBI prevalence and annual incidence [20]. A TB contact investigation study from Korea showed that the LTBI rate was 26.2% among contacts aged ≥35 years, compared with 5.7% among those aged 15–19 years [24]. Another study from Korean tertiary hospitals using a QuantiFERON-TB Gold assay showed findings similar to those of our study. The annual IGRA seroconversion rate ranged from 0.7% to 1.5% over a five-year study period, and seroconverters were older than non-converters (median age, 42 vs. 38 years; *p* = 0.02); however, high-risk group classification was not associated with seroconversion [25]. The association between increasing age and LTBI seroconversion likely reflects both cumulative exposure burden and age-related changes in T-cell immunity. Given that acquired LTBI depends on effective containment of *Mycobacterium tuberculosis* by T-cell-mediated immune responses, alterations in cellular immune function with aging may increase susceptibility to infection even under comparable exposure conditions [26]. This interpretation is supported by experimental and human data demonstrating that control of *M. tuberculosis* depends on the balance between bacterial burden and the timing and effectiveness of T-cell-mediated immunity. Repeated or sustained exposure may overwhelm host immune containment, while age-related alterations in cellular immune function can further increase susceptibility to LTBI [27].

This study has several limitations. First, this was a single-center study conducted at a tertiary-care hospital, and caution is therefore warranted when generalizing the findings to settings with different tuberculosis epidemiology, healthcare systems, or infection control practices. For example, in countries with low TB incidence and well-established isolation infrastructure, TB exposure may be evaluated without routine periodic LTBI follow-up, which could limit the applicability of our findings to such settings. Second, the overall LTBI seroconversion rate was relatively low. Although we applied appropriate statistical methods to address sparse outcome events, including penalized regression techniques, the limited number of seroconversion events may have constrained the precision of effect estimates. Third, although the severity of TB exposure was defined using clear institutional criteria based on national guidelines, healthcare workers were required to wear N95-equivalent personal protective equipment during exposure events. As a result, all documented exposures were classified as casual contacts, and detailed information regarding other qualitative aspects of exposure—such as proximity, ventilation conditions, or intensity of contact—was not available beyond exposure frequency. In addition, the routine use of PPE among healthcare workers may have attenuated the risk of transmission, potentially leading to an underestimation of the observed T-SPOT.TB seroconversion rate (1.0%) in this study. Finally, the observation period was relatively short, spanning approximately one year, which may limit the ability to capture longer-term LTBI acquisition patterns. Multicenter studies with extended follow-up durations are therefore needed to validate and extend these findings.

## 5. Conclusions

In conclusion, occupational TB risk group classification was useful for predicting the frequency of TB exposure among healthcare workers. However, LTBI occurrence was not clearly differentiated by occupational risk groups and appeared to be more closely associated with individual-level factors, including cumulative TB exposure and age. These findings suggest that uniform periodic LTBI screening strategies based solely on occupational risk groups classification may have limitations. An individualized approach that considers cumulative patient exposure—even in the absence of documented close contact—as well as demographic factors such as age, may help to better identify healthcare workers at increased risk of LTBI acquisition.

## Figures and Tables

**Table 1:** Latent Tuberculosis Infection Screening for Healthcare Workers Strength and Timing of Recommendations by KDCA.

Risk Group		Relevant Departments	Initial Screening	Periodic Screening
Group 1	Personnel involved in the examination, diagnosis, treatment, or nursing care of TB patients (including healthcare professionals, medical technologists, and nursing assistants)	✓ Pulmonology outpatient clinics and wards ✓ Bronchoscopy units ✓ Mycobacteriology (tuberculosis) laboratories ✓ Pulmonary function testing laboratories ✓ Infectious Diseases outpatient clinics and wards ✓ Pulmonology outpatient clinics and wards ✓ Intensive Care Units (ICUs) ✓ Emergency Departments ✓ Chest imaging/radiology departments	Required	Required
Group 2	Personnel in close contact with high-risk patients (e.g., neonates or immunocompromised individuals)	✓ Neonatal units and Neonatal Intensive Care Units ✓ Pediatric outpatient clinics ✓ Obstetrics and Gynecology outpatient clinics and wards ✓ Delivery rooms ✓ Hematology–Oncology outpatient clinics and wards ✓ Dialysis units ✓ Administrative departments with direct patient contact (e.g., patient administration or registration offices) ✓ Personnel who have worked in Group 1 departments for ≥1 year and are subsequently reclassified into Group 2 for the following year	Required	Highly recommended
Group 3	Personnel with possible exposure to airborne infections	✓ Dental clinics ✓ Thoracic surgery outpatient clinics and wards ✓ Anesthesiology departments	Required	Highly recommended
Group 4	Other clinical or support staff where standard precautions apply	✓ Clinical healthcare workers not classified into Groups 1–3 ✓ Environmental cleaning staff ✓ Patient transport personnel	Required	Recommended
Group 5	Other healthcare facility personnel with low exposure risk	✓ Administrative staff with minimal or no patient contact	Required	Not recommended

All healthcare workers are required to undergo baseline LTBI screening, while the intensity of periodic screening is determined according to occupational TB exposure risk.

**Table 2:** Differences in Tuberculosis Exposure Across Occupational Risk Groups in Healthcare Workers.

Risk Group	Overall (2116)	Group 1 (542)	Group 2 (400)	Group 3 (827)	Group 4 (171)	Group 5 (176)	*p*-Value
Sex							<0.001
Male	587 (27.7)	151 (27.9)	60 (15.0)	220 (26.6)	54 (31.6)	102 (58.0)	
Female	1529 (72.3)	391 (72.1)	340 (85.0)	607 (73.4)	117 (68.4)	74 (42.0)	
Age (years), median (interquartile range)	33 (27–44)	30 (27–41)	31 (26–45)	33 (27–43)	37 (30–49)	40 (32–48)	<0.001
Tuberculosis Exposure							<0.001
No	1581 (74.7)	288 (53.1)	275 (68.8)	674 (81.5)	169 (98.8)	176 (99.6)	
Yes	535 (25.3)	254 (46.9)	125 (31.3)	153 (18.5)	2 (1.2)	1 (0.6)	
Number of tuberculosis exposures per person, (/person), mean ± standard deviation	0.62 ± 1.30	1.39 ± 1.88	0.74 ± 1.29	0.31 ± 0.77	0.01 ± 0.11	0.01 ± 0.07	<0.001

Data are presented as *n* (%) unless otherwise stated; All *p*-values represent overall comparisons across the five occupational risk groups (Groups 1–5). Categorical variables were compared using the chi-square test, and continuous variables were compared using the Kruskal–Wallis test.

**Table 3:** Associated Factors With T-SPOT.TB Seroconversion by Occupational TB Risk Group in Healthcare Workers (*n* = 1323).

Variables		Univariable Analysis			Multivariable Analysis	
		LTBI (*n* = 13)	Non-LTBI (*n* = 1310)	*p*-Value	Adjusted OR (95% CI)	*p*-Value
Age, years	31 (27–41)	49 (26–56)	31 (27–41)	0.115	1.09 (1.03–1.15)	0.001
Sex				0.320		0.057
Male	286 (21.6)	1 (7.7)	285 (21.8)		Reference	
Female	1037 (78.4)	12 (92.3)	1025 (78.2)		4.47 (0.96–44.33)	
Risk group				0.904		
Group 1	354 (26.8)	3 (23.1)	351 (26.8)		Reference	
Group 2	296 (45.5)	2 (15.4)	294 (22.4)		1.00 (0.16–5.39)	0.998
Group 3	573 (65.9)	7 (53.8)	566 (43.2)		1.94 (0.49–9.23)	0.354
Group 4	100 (14.9)	1 (7.7)	99 (7.7)		2.01 (0.16–17.20)	0.546
Tuberculosis exposure observation period (days)	371 (350–392)	370 (366.0–498.0)	371 (350.0–392.0)	0.266	1.01 (1.00–1.01)	0.090
Exposure frequency				0.018		
0	990 (74.8)	7 (53.8)	983 (75.0)		Reference	
1	158 (11.9)	1 (7.6)	157 (12.0)		1.62 (0.16–8.13)	0.619
≥2	175 (13.2)	5 (38.4)	170 (13.0)		7.03 (1.87–26.36)	0.005

LTBI, latent tuberculosis infection; OR, odds ratio; CI, confidence interval. Data are presented as n (%) or median (interquartile range), unless otherwise stated. Percentages represent the distribution within each outcome group. Categorical variables were compared using Fisher’s exact test. Multivariable analysis was performed using Firth’s penalized logistic regression.

## Data Availability

The raw data supporting the conclusions of this article will be made available by the authors on request.

## Abbreviations

The following abbreviations are used in this manuscript:

LTBI	Latent tuberculosis infection
TB	Tuberculosis
TST	Tuberculin skin test
IGRA	Interferon-γ release assays
BCG	Bacille Calmette–Guérin
SFC	Spot-forming cells
PPE	Personal protective equipment
HCW	healthcare worker
